# The Role of High-Density Lipoprotein, C-reactive Protein, and Serum Ferritin in Ischemic and Hemorrhagic Stroke: An Observational Cross-Sectional Comparative Study

**DOI:** 10.7759/cureus.66606

**Published:** 2024-08-10

**Authors:** Mohith Prakash Kondapalli, Govind Shiddapur, Nilesh Jagdale, Vutukuru Kalyan Kumar Reddy, Saimounika Adapa

**Affiliations:** 1 Department of General Medicine, Dr. D. Y. Patil Medical College, Hospital and Research Centre, Dr. D. Y. Patil Vidyapeeth (Deemed to be University), Pune, IND

**Keywords:** hemorrhagic stroke, ischemic stroke, serum ferritin, c-reactive protein (crp), high-density lipoprotein (hdl)

## Abstract

Background

Stroke is a significant global health issue, with a high prevalence of morbidity, mortality, and disability. We can classify strokes into two types: ischemic and hemorrhagic, with ischemic strokes being more common. This study aims to investigate the role of high-density lipoprotein (HDL), C-reactive protein (CRP), and serum ferritin levels in people who have had ischemic and hemorrhagic strokes in order to identify possible biomarkers for diagnosis and treatment.

Materials and methods

This observational cross-sectional comparative study included 100 stroke patients (50 ischemic and 50 hemorrhagic) from Dr. D. Y. Patil Medical College, Hospital and Research Centre, Dr. D. Y. Patil Vidyapeeth (Deemed to be University), Pune. We collected data through clinical evaluations, laboratory tests, and imaging studies. We measured and analyzed HDL, CRP, and serum ferritin levels using appropriate statistical tests, such as the chi-square test and Student t-test, with a 95% confidence interval (CI) and a 5% p-value for significance.

Results

The mean age for ischemic stroke patients was 55.92 years, whereas for hemorrhagic stroke patients, it was 58.68 years. The study found significant differences in HDL, CRP, and ferritin levels between the two groups. The mean HDL level for ischemic stroke patients was significantly lower at 25.10 mg/dL, compared to 40.57 mg/dL in hemorrhagic stroke patients, with a p-value of <0.001. The mean CRP level was higher in ischemic stroke patients (28.90 mg/L) compared to hemorrhagic stroke patients (22.80 mg/L), with a p-value of <0.001. Ferritin levels were also higher in hemorrhagic stroke patients (587.98 ng/mL) compared to ischemic stroke patients (473.16 ng/mL), with a statistically significant p-value of <0.001.

Conclusion

This study highlights the significant role of HDL, CRP, and serum ferritin levels in distinguishing between ischemic and hemorrhagic stroke patients. Elevated HDL levels may protect against ischemic strokes due to their anti-inflammatory properties, while higher CRP levels in ischemic strokes indicate a strong inflammatory response. Elevated ferritin levels in hemorrhagic strokes suggest increased oxidative stress and inflammation.

## Introduction

Stroke is a global health issue. It is a prevalent medical crisis that significantly impacts the rates of illness, death, and disability in both industrialized and developing nations. The prevalence increases significantly with advancing age, and in numerous emerging nations, the prevalence is on the rise due to the adoption of less healthful behaviors. Approximately 20% of individuals who experience an acute stroke will succumb to the condition within one month, while at least 50% of those who survive will be left with physical impairment. Stroke is the abrupt demise of brain cells caused by insufficient blood supply. However, the visible manifestations of a stroke do not consistently reveal the precise cause or causes underlying them. Common indicators of a stroke encompass the abrupt onset of unilateral paralysis, vision impairment, speech difficulties, memory decline, cognitive impairment, loss of consciousness, or fatality [1].

It can be broadly categorized into two main types:

Ischemic stroke: This type occurs when there is a restriction or interruption of blood supply to a part of the brain, typically due to an obstruction in a blood vessel. Causes of ischemic stroke include thrombosis, embolism, systemic hypoperfusion, and venous thrombosis. Cryptogenic strokes, where the cause is unknown, account for about 30-40% of ischemic strokes [2, 3].

Hemorrhagic stroke: This type involves bleeding within the brain caused by the rupture of a blood vessel or an abnormal vascular structure. Hemorrhagic strokes can be further classified into intracerebral and subarachnoid strokes. In terms of prevalence, approximately 88% of all strokes are ischemic, while the remaining 12% are hemorrhagic. Among hemorrhagic strokes, 9% are because of intracranial hemorrhage, and 3% are because of subarachnoid hemorrhage. Differentiating between hemorrhagic and ischemic strokes is crucial for effective management and treatment decisions [3].

High-density lipoprotein (HDL) cholesterol in stroke pathogenesis is significant due to its cardioprotective effects, including reverse cholesterol transport, anti-inflammatory properties, and endothelial function improvement. Low HDL levels are linked to increased ischemic stroke risk, possibly due to promoting atherosclerosis and possessing anti-thrombotic properties [4].

Conversely, higher HDL levels might protect against hemorrhagic stroke through anti-inflammatory and endothelial protection, although excessively high levels could increase hemorrhagic stroke risk by altering blood viscosity and hemostatic factors [5]. Understanding HDL's role in both stroke types is crucial for targeted prevention and treatment strategies.

C-reactive protein (CRP) is a marker of inflammation in the body. CRP levels rise in response to systemic inflammation and are linked to both ischemic and hemorrhagic strokes. Elevated CRP levels are predictive of stroke risk and poorer outcomes, reflecting the degree of inflammation. The mechanisms by which CRP contributes to stroke include impairing endothelial function, promoting plaque instability, and activating leukocytes, further exacerbating the inflammatory process. Measuring CRP levels can aid in assessing stroke risk and guiding treatment decisions, highlighting its importance as both a biomarker and therapeutic target for better stroke management and prevention [6].

Serum ferritin is a blood protein that stores iron and releases it in a controlled way. It serves as an indicator of the body's iron stores. Elevated serum ferritin levels can indicate iron overload, which has been linked to a variety of health issues, including stroke. High ferritin levels may contribute to oxidative stress and inflammation, which can damage blood vessels and increase stroke risk [7].

Aims and objectives

The study aims to analyze and compare the levels of HDL, CRP, and serum ferritin in patients diagnosed with ischemic and hemorrhagic strokes. Initially, we diagnose and classify patients into either ischemic or hemorrhagic stroke groups. We then measure the levels of HDL, CRP, and serum ferritin in both groups. The final objective is to compare these levels between patients with ischemic and hemorrhagic strokes to identify any significant differences or patterns.

## Materials and methods

Study design

The study conducted at Dr. D. Y. Patil Medical College, Hospital and Research Centre, Dr. D. Y. Patil Vidyapeeth (Deemed to be University), Pune, from September 2022 to June 2024, is an observational cross-sectional comparative study involving a sample size of 100 cases. Prior to commencing the research, ethical approval was obtained from the institutional ethics committee (ethical committee clearance number: IESC/PGS/2022/10). All patients participating in the study provided written informed consent. The data collection process included several components: patients underwent both routine and specialized laboratory investigations. Additionally, demographic information, such as age and sex, along with histories of comorbid conditions and presenting complaints, were recorded through patient interviews. Furthermore, a detailed neurological clinical examination was conducted via physical assessment and appropriate diagnostic tests. All findings were systematically documented using a pre-designed and pre-tested proforma. This structured approach ensured comprehensive data gathering to facilitate the study's comparative analysis within the specified timeframe and research setting.

Inclusion and exclusion criteria

The study at Dr. D. Y. Patil Medical College, Hospital and Research Centre, Dr. D. Y. Patil Vidyapeeth (Deemed to be University), Pune, focused on patients meeting specific inclusion and exclusion criteria. The inclusion criteria comprised individuals aged over 18 years with a radiologically confirmed ischemic or hemorrhagic stroke within 24 hours. Exclusion criteria included patients with space-occupying lesions, underlying liver or renal pathology, those on lipid-lowering therapy, individuals with familial dyslipidemia, and patients receiving recurrent blood transfusions. Key investigations conducted as part of the study included measuring CRP levels (normal level: <10 mg/L), serum ferritin levels (normal range: 21.8-274.66 ng/mL), assessing HDL levels, and performing radio imaging. These criteria and investigations were essential for characterizing and analyzing the relationship between lipid profiles, inflammatory markers, and stroke types within the specified patient population and study period.

Sample size

The study by Sarika et al. [8], "High Sensitivity C-reactive Protein Levels and Lipid Profile in Ischemic and Hemorrhagic Stroke," examined the mean and standard deviation (SD) of HDL levels in ischemic stroke (39.37 + 7.1 mg/dL) and hemorrhagic stroke (44.47 + 7.1 mg/dL), respectively, with a significance level of 0.05 and a power of 0.80, a sample size of 31 participants per each group is required. However, we chose to take 50 participants from each group. WinPepi version 11.65 (Joseph H. Abramson, Jerusalem, Israel) was the software used.

Data collection and consent

The purpose of this study was to investigate and compare the role of HDL, CRP, and serum ferritin levels in ischemic and hemorrhagic strokes. Participants were recruited, and their involvement included a thorough review of their medical history and a series of clinical and laboratory investigations. Data collection involved patient interviews to gather demographic information and medical history, physical examinations for neurological assessment, and specialized laboratory investigations such as HDL, CRP, and serum ferritin.

Participants or their guardians were given comprehensive information regarding the study, encompassing its objectives, methodologies, potential risks, and advantages. The participants were notified that their involvement was optional and that they had the option to withdraw at any point without any consequences for their medical treatment. The study ensured participants' data confidentiality and refrained from disclosing their identities in any resulting publications. Every participant was obligated to sign a permission form confirming that they had perused and comprehended the offered material, had the chance to inquire about any uncertainties, and consented to take part in the study. This study aims to improve our understanding of the function of HDL, CRP, and serum ferritin in ischemic and hemorrhagic stroke by collecting extensive data on medical history, clinical examination results, and pertinent blood investigations.

Statistical analysis

All of the collected information was input into a spreadsheet (Microsoft Excel 2010, Microsoft® Corp., Redmond, WA) and then transferred to the data editor in SPSS version 20 (IBM SPSS Statistics for Windows, IBM Corp., Armonk, NY). Percentages, averages, and SDs were all computed as part of the descriptive statistics. The study made use of the Student t-test and chi-square test for statistical significance. Specifically, the confidence interval (CI) and p-value were established at 95% and 5%, respectively.

## Results

The comparative analysis of the mean age of patients diagnosed with ischemic and hemorrhagic strokes is depicted in Table 1. The mean age of ischemic stroke patients is 55.92 years, indicating that this type of stroke predominantly affects middle-aged and older adults. In contrast, the mean age for hemorrhagic stroke patients is slightly higher at 58.68 years. By using the Student-t test, a p-value of 0.357 suggests that there is no significant difference in the average age between the two groups.

**Table 1:** Age distribution in both the groups

Stroke type	Mean age in years
Ischemic stroke	55.92
Hemorrhagic stroke	58.68
p-value	0.357

The mean HDL level for ischemic stroke patients is significantly lower at 25.10 mg/dL with an SD of 9.66 and a 95% CI ranging from 22.40 to 27.80 mg/dL. In contrast, hemorrhagic stroke patients exhibit a higher mean HDL level of 40.57 mg/dL with an SD of 13.43 and a 95% CI for ischemic stroke patients ranging from 36.49 to 44.99 mg/dL, also suggesting precise estimates. By using the Student t-test, a p-value for the difference in mean HDL levels between the two stroke types is <0.001, indicating that this difference is statistically significant (Table 2).

**Table 2:** HDL levels in both the groups CI: confidence interval; HDL: high-density lipoprotein; mg/dL: milligrams per deciliter; SD: standard deviation

Stroke type	Mean HDL (mg/dL)	SD	95% CI lower bound	95% CI upper bound	p-value
Hemorrhagic stroke	40.57	13.43	36.49	44.99	<0.001
Ischemic stroke	25.10	9.66	22.40	27.80	

The mean CRP level for patients with ischemic stroke is significantly higher at 28.90 mg/L with an SD of 7.48 and a 95% CI ranging from 26.79 to 31.01 mg/L. In contrast, hemorrhagic stroke patients exhibit a lower mean CRP level of 22.80 mg/L with an SD of 10.97, and a 95% CI for ischemic stroke patients ranges from 19.75 to 25.85 mg/L. By using the Student-t test, a p-value for the difference in mean CRP levels between the two stroke types is less than 0.001, indicating that this difference is statistically significant (Table 3).

**Table 3:** CRP levels in both the groups CI: confidence interval; CRP: C-reactive protein; mg/L: milligrams per liter; SD: standard deviation

Stroke type	Mean CRP (mg/L)	SD	95% CI lower bound	95% CI upper bound	p-value
Hemorrhagic stroke	22.80	10.97	19.75	25.85	<0.001
Ischemic stroke	28.90	7.48	26.79	31.01	

The mean ferritin level of ischemic stroke patients is 473.16 ng/mL, with an SD of 138.57 and a 95% CI ranging from 432.35 to 514.00 ng/mL. In contrast, hemorrhagic stroke patients exhibit a higher mean ferritin level of 587.98 ng/mL, with an SD of 156.02 and a 95% CI between 540.23 and 635.73 ng/mL. A statistically significant difference in ferritin levels between the two groups is shown by a p-value of <0.001 by using the Student-t test (Table 4).

**Table 4:** Serum ferritin levels in both the groups CI: confidence interval; ng/mL: nanograms per milliliter; SD: standard deviation

Stroke type	Mean serum ferritin (ng/mL)	SD	95% CI lower bound	95% CI upper bound	p-value
Ischemic stroke	473.16	138.57	432.35	514.00	<0.001
Hemorrhagic stroke	587.98	156.02	540.23	635.73	

## Discussion

This study focuses on the comparison of age, serum HDL, CRP, and serum ferritin levels in ischemic and hemorrhagic stroke patients, providing valuable insights into their roles as biomarkers in stroke prognosis.

Analysis of the data reveals that the mean age of patients with ischemic stroke is 54.96 years, with an SD of 11.10 years. In contrast, patients with hemorrhagic stroke have a mean age of 56.7 years and an SD of 7.31 years. The p-value of 0.357 suggests that there is no significant difference in the average age between the two groups. Andersen et al. [9] reported a mean age of 72.7 years (SD = 12.2) for ischemic stroke patients and 72.9 years (SD = 12.1) for hemorrhagic stroke patients, with a p-value of 0.258. These results indicate no significant difference in the average age between individuals with ischemic and hemorrhagic strokes. Both studies consistently show that the mean age of patients with ischemic and hemorrhagic strokes is not significant, even if the age ranges observed in the two studies are different.

The current study, along with the findings by Vakilian et al. [10] and Saproo et al. [11], provides a comprehensive comparison of HDL cholesterol levels in patients with hemorrhagic and ischemic strokes. The current study demonstrates that individuals with hemorrhagic stroke exhibit markedly elevated levels of HDL cholesterol in comparison to those with ischemic stroke. Specifically, the mean HDL level for patients with hemorrhagic stroke is 40.57 mg/dL, with an SD of 13.43. The CI for hemorrhagic stroke patients ranges from 36.49 to 44.99 mg/dL, indicating precise estimates with a 95% level of confidence. Conversely, those who have experienced an ischemic stroke have a notably lower average HDL level of 25.10 mg/dL, with an SD of 9.66. The 95% CI for ischemic stroke patients is 22.40 to 27.80 mg/dL. The p-value for the difference in average HDL levels between the two stroke types is less than 0.001, underscoring a statistically significant disparity.

Vakilian et al. [10] corroborate these findings in their study, where the mean HDL level was significantly higher in hemorrhagic stroke patients (42.55 mg/dL) compared to ischemic stroke patients (36.86 mg/dL). The p-value of 0.007 further confirms the statistical significance of this difference. This study aligns with the current study’s findings, reinforcing the pattern that higher HDL levels are associated with hemorrhagic strokes, whereas lower HDL levels are prevalent in ischemic strokes. This difference in HDL levels could be indicative of the underlying pathophysiological differences between the two types of strokes, highlighting the protective role of HDL in cardiovascular health and its varying impact on different cerebrovascular events.

Similarly, the study conducted by Saproo et al. [11], with 50 cases each of ischemic and hemorrhagic strokes, also found significant differences in HDL levels between the two groups. The mean HDL level for hemorrhagic stroke patients was 39.6 ± 7.8 mg/dL, whereas for ischemic stroke patients, it was 36.2 ± 6.3 mg/dL. This difference, with a p-value of 0.034, signifies a statistically significant higher HDL level in hemorrhagic stroke patients. It was concluded that there is a strong link between abnormal HDL levels and ischemic stroke, indicating that lipid profile abnormalities, especially lower HDL levels, are more common in ischemic stroke patients. This comparison among the three studies consistently points toward a distinct lipid profile characteristic of hemorrhagic versus ischemic stroke patients, emphasizing the importance of HDL levels as a differential biomarker in stroke management and prognosis.

The current study reports that ischemic stroke patients exhibit significantly higher CRP levels, with a mean of 28.90 mg/L, an SD of 7.48, and a 95% CI ranging from 26.79 to 31.01 mg/L. In contrast, hemorrhagic stroke patients have a lower mean CRP level of 22.80 mg/L with a broader SD of 10.97 and a 95% CI of 19.75 to 25.85 mg/L. The p-value for the difference in mean CRP levels between the two stroke types is less than 0.001, indicating a statistically significant disparity. These findings suggest that ischemic strokes are associated with a higher inflammatory response compared to hemorrhagic strokes. In a comparative study by Roudbary et al. [12], ischemic stroke patients had a mean CRP level of 18.92 ± 11.28 mg/L, while hemorrhagic stroke patients exhibited a substantially lower mean CRP level of 2.65 ± 1.70 mg/L. This significant difference, with a p-value of less than 0.0001, supports the observation that ischemic stroke induces a more pronounced inflammatory response than hemorrhagic stroke. The substantial variance in CRP levels among ischemic stroke patients across different studies might reflect the diversity in stroke severity and the presence of underlying conditions. These findings emphasize the importance of CRP as a marker of inflammation and its correlation with stroke severity and prognosis. Managing inflammation through targeted therapies could be crucial for improving outcomes in stroke patients, highlighting the need for further research into the inflammatory pathways involved in different types of strokes.

According to this study, ischemic stroke patients have a mean ferritin level of 473.16 ng/mL, with an SD of 138.57 and a 95% CI ranging from 432.35 to 514.00 ng/mL. In contrast, hemorrhagic stroke patients exhibit a higher mean ferritin level of 587.98 ng/mL, with an SD of 156.02 and a 95% CI between 540.23 and 635.73 ng/mL. The p-value of <0.001 indicates a statistically significant difference in ferritin levels between the two groups. This study's findings align with Mythreini et al.'s research [13]. Their study showed that serum ferritin levels were significantly higher in patients with hemorrhagic strokes than in patients with ischemic strokes (p = 0.010). Ferritin serves as an acute-phase reactant, indicating both iron storage levels and inflammatory burden. Higher ferritin levels in hemorrhagic stroke patients may reflect greater oxidative stress and inflammation, contributing to the pathophysiology of brain hemorrhage. It also demonstrated that elevated ferritin levels are associated with poorer outcomes and increased severity in hemorrhagic stroke patients. The findings underscore the potential role of ferritin as a biomarker for stroke severity and prognosis, particularly in hemorrhagic stroke.

Limitations

The study has a few limitations that should be considered when interpreting the results. Firstly, the sample size of 100 patients, while providing valuable initial insights, is relatively small and may not capture the full spectrum of variability in biomarker levels among stroke patients. This limits the findings' generalizability to a larger population. Secondly, the study’s cross-sectional design inherently restricts the ability to establish causality between the observed biomarker levels (HDL, CRP, and ferritin) and stroke outcomes. Longitudinal studies are needed to determine whether these biomarkers can predict stroke prognosis over time. Lastly, the study was conducted at a single medical center, which may introduce a selection bias and limit the external validity of the results. Future research should include larger, more diverse populations across multiple centers to validate these findings and ensure they are representative of the broader stroke patient population.

## Conclusions

This study underscores the significant role of HDL, CRP, and serum ferritin levels in differentiating between ischemic and hemorrhagic stroke patients. The findings highlight the potential of these biomarkers for stroke prognosis and management. Elevated HDL levels appear to have a protective role in preventing ischemic strokes, likely due to their anti-inflammatory and endothelial-protective properties. On the other hand, higher CRP levels indicate a pronounced inflammatory response in ischemic stroke, emphasizing inflammation's role in its pathophysiology. Additionally, elevated ferritin levels in hemorrhagic stroke patients suggest greater oxidative stress and inflammation, contributing to the severity of brain hemorrhage. These differences in biomarker levels between stroke types suggest that HDL, CRP, and ferritin can serve as valuable tools not only for differential stroke diagnosis but also for understanding the underlying pathophysiological processes. This understanding can guide more targeted and effective therapeutic strategies. We need to conduct further research with larger and more diverse populations to validate these findings and fully establish the clinical utility of these biomarkers in stroke management.
